# Serological investigation of plague and brucellosis infection in *Marmota himalayana* plague foci in the Altun Mountains on the Qinghai-Tibet Plateau

**DOI:** 10.3389/fpubh.2022.990218

**Published:** 2022-11-18

**Authors:** Shuai Qin, Junrong Liang, Deming Tang, Yuhuang Chen, Ran Duan, Xinmin Lu, Asaiti Bukai, Xiaojin Zheng, Dongyue Lv, Zhaokai He, Weiwei Wu, Haonan Han, Huaiqi Jing, Xin Wang

**Affiliations:** ^1^State Key Laboratory of Infectious Disease Prevention and Control, National Institute for Communicable Disease Control and Prevention, Chinese Center for Disease Control and Prevention, Beijing, China; ^2^Shenzhen Nanshan Maternity and Child Healthcare Hospital, Shenzhen, China; ^3^Akesai Kazak Autonomous County Center for Disease Control and Prevention, Jiuquan, China

**Keywords:** plague, brucellosis, seroprevalence, *Marmota himalayana* plague foci, previous infection

## Abstract

The Altun Mountains are among the most active regions of *Marmota himalayana* plague foci of the Qinghai-Tibet Plateau where animal plague is prevalent, whereas only three human cases have been found since 1960. Animal husbandry is the main income for the local economy; brucellosis appears sometimes in animals and less often in humans. In this study, a retrospective investigation of plague and brucellosis seroprevalence among humans and animals was conducted to improve prevention and control measures for the two diseases. Animal and human sera were collected for routine surveillance from 2018 to 2021 and screened for plague and brucellosis. *Yersinia pestis* F1 antibody was preliminarily screened by the colloidal gold method at the monitoring site to identify previous infections with positive serology. Previous plague infection was found in 3.2% (14/432) of the studied human population having close contact with livestock, which indicates evidence of exposure to the *Yersinia* antigen (dead or live pathogenic materials) in the Altun Mountains. Seroprevalence of brucellosis was higher in camels (6.2%) and sheepdogs (1.8%) than in other livestock such as cattle and sheep, suggesting a possible transmission route from secondary host animals to humans.

## Introduction

Brucellosis and plague are both natural focus diseases that are separately recognized as neglected diseases and re-emerging diseases by the World Health Organization (1–3). With the economic globalization and rapid development of the transportation industry, the possibility of occurrence of imported cases in non-endemic foci is increasing (4–8). *Marmota himalayana* plague foci of the Qinghai-Tibet Plateau are the most active foci in China, whereas the Alutun Mountains are the most active region (9, 10). In the *M. himalayana* plague focus in China before the 1990s, most human cases occurred here. Since the 1990s, rat-associated plague epidemics have erupted in southern China, but beginning in 2004, the *M. himalayana* plague focus re-emerged as the main source of human cases. Outbreaks have occurred here every few years (11). Each year, *Y. pestis* is isolated in a number of marmots found dead in the environment (12). However, only three human cases have been found since 1960 (13). The reason for this paradox is not known. Brucellosis is also an important zoonosis in the Altun Mountains where animal husbandry is practiced (14). In 2020, a brucellosis outbreak occurred in camel herd. The local transmission of brucellosis was of concern. In this study, the findings of previous plague infection in humans and transmission of brucellosis from a secondary host can help improve the prevention and control of these two significant zoonoses (15).

## Materials and methods

**Figure 1:**
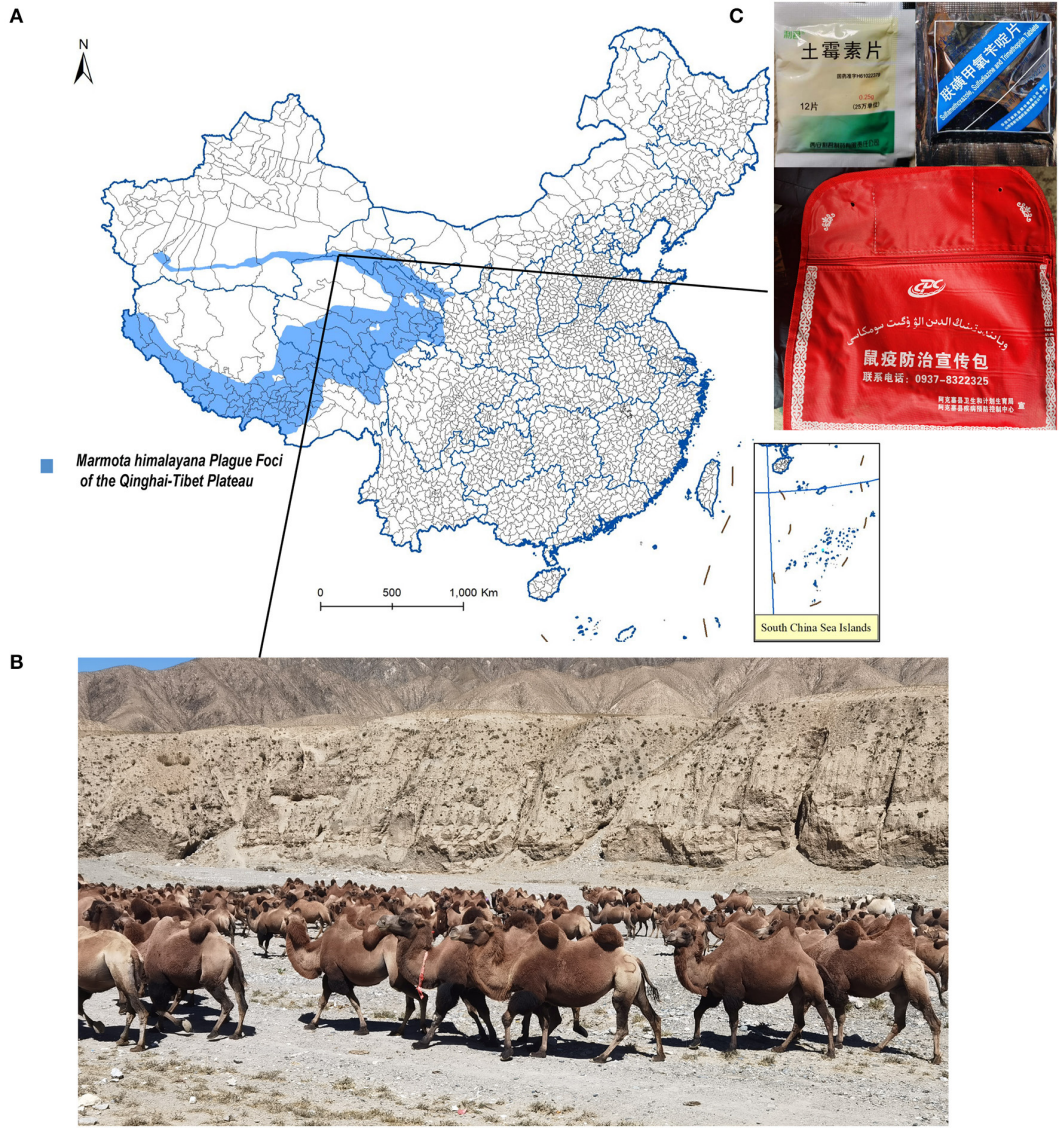
Ecology of the studied region and plague first-aid kit given to herdsmen. **(A)** Geographic region of this study. **(B)** Landscape of camel's living habitat. **(C) (Left)** Tablet oxytetracycline contained in the Plague first-aid kit. (Right) Sulfamethoxazole, sulfadiazine, and trimethoprim contained in the Plague first-aid kit. **(Bottom)** Plague first-aid kit for herdsmen.

The Altun Mountains located on the north of the *M. himalayana* plague foci of the Qinghai-Tibet Plateau (Figure 1A) are mainly desert and semi-desert grasslands (Figure 1B). The area where the residents live is vast and sparsely populated (10,000 people in 31,000 km^2^). Among the livestock raised, the number of sheep is the largest, which is about 120,000 per year. That of cattle, horses, camels, and other large livestock is about 6,000 per year. Free-ranging assisted by sheepdogs is the main husbandry pattern. *M. himalayana* are plentiful in number, widely distributed, have a high natural carrier rate of *Y. pestis*, and are the foci's main reservoir of *Y. pestis* (16).

This retrospective study was conducted by the National Institute for Communicable Disease Control and Prevention, Chinese Center for Disease Control and Prevention. To analyze the average seroprevalence levels of plague and brucellosis, at least two years of human and animal samples collected as part of routine surveillance were included in this study. Based on the screening test adopted by the laboratory at the monitoring site, the positive samples were further confirmed by the superior laboratory.

Blood samples were collected and sera were separated by centrifugation and frozen at −80°C. Serological monitoring of brucellosis in livestock (camel, cattle, sheep, and sheepdogs) and persons whose occupations were breeder, herder, veterinarian, and other occupations that were in close contact with livestock was carried out. The serum samples were collected during routine surveillance of plague and brucellosis from 2018 to 2021. The gender, age group (17), and occupation information was also collected. Human sera collected for brucellosis surveillance was also tested for the plague. The sera of marmots collected for plague surveillance was both tested for plague and brucellosis.

The colloidal gold method was used for screening for *Y. pestis* F1 antibodies (Beijing Jianaixi Biotechnology Co., Ltd., Beijing, China) and the indirect hemagglutination assay was performed for verification (Qinghai Province Endemic Disease Prevention and Control Institute, Xining, Qinghai Province, China). F1 antigen inhibition controls, negative controls, and positive controls were established. An antibody titer ≥ 1:16 was identified as positive.

The rose bengal plate test was used for screening for brucella antibodies (Idexx Laboratories, Westbrook, Maine, United States; Lanzhou Institute of Biological Products Co., Ltd., Lanzhou, Gansu Province, China). A total of 30 μL antigen and 30 μL serum samples were mixed on a flat plate. Results were read immediately after 4 min. Positive samples were further tested by Wright's serum agglutination tests (Idexx Laboratories, Westbrook, Maine, United States). Sera were diluted by 1:5, 1:10, 1:20, 1:40, 1:80, and 1:160, and then equal-volume brucellosis antigen was added to each tube. Tubes were thoroughly mixed and incubated for 18–20 h at 37 ± 3°C. Turbidimetric tubes were used to compare the serum agglutination degree of samples. Samples ≥30 IU/ml were identified as positive. The antigen used in the study can detect *B. suis, B. melitensis*, and *B. abortus*.

Statistical analysis was conducted to compare seroprevalence among different groups (SPSS Version 26.0). According to specific theoretical frequency, Pearson's chi-square test (T ≥ 35), Yates's continuity correction (1 ≤ T < 5), or Fisher's exact test (T < 1) was applied to assess associations between variables of concern and the seroprevalence of brucellosis or plague.

## Results

A total of 432 individuals between ages of 7 and 70 with certain occupations that were in close contact with livestock from January 2020 to July 2021 were tested for brucella and *Y. pestis*. The sample population was engaged in animal husbandry, including breeders (168), herders (167) veterinarians (59), and other occupations, including 38 individuals who purchase, process, or sale livestock products, such as fur, milk, meat, etc. A total of 5,799 livestock serum samples were tested for brucella. Samples included sera from cattle (987), camels (3,820), and sheep (882) collected from 2019 to 2020, and sheepdogs (110) collected from January 2020 to July 2021. To analyze the average seroprevalence level of brucellosis, human and dog samples collected in 2020 and 2021 were included because samples collected in 2019 were not available. No positive marmots were detected in the same period, so the range of detection years was expanded. A total of 360 marmot sera samples collected from January 2018 to July 2021 were tested for brucella and *Y. pestis* antibodies (because of insufficient sample volume, 73 marmot samples collected from 2018 to 2019 were tested only for brucella, while 287 marmot samples collected from January 2020 to July 2021 were tested for brucella and *Y. pestis*).

**Figure 2:**
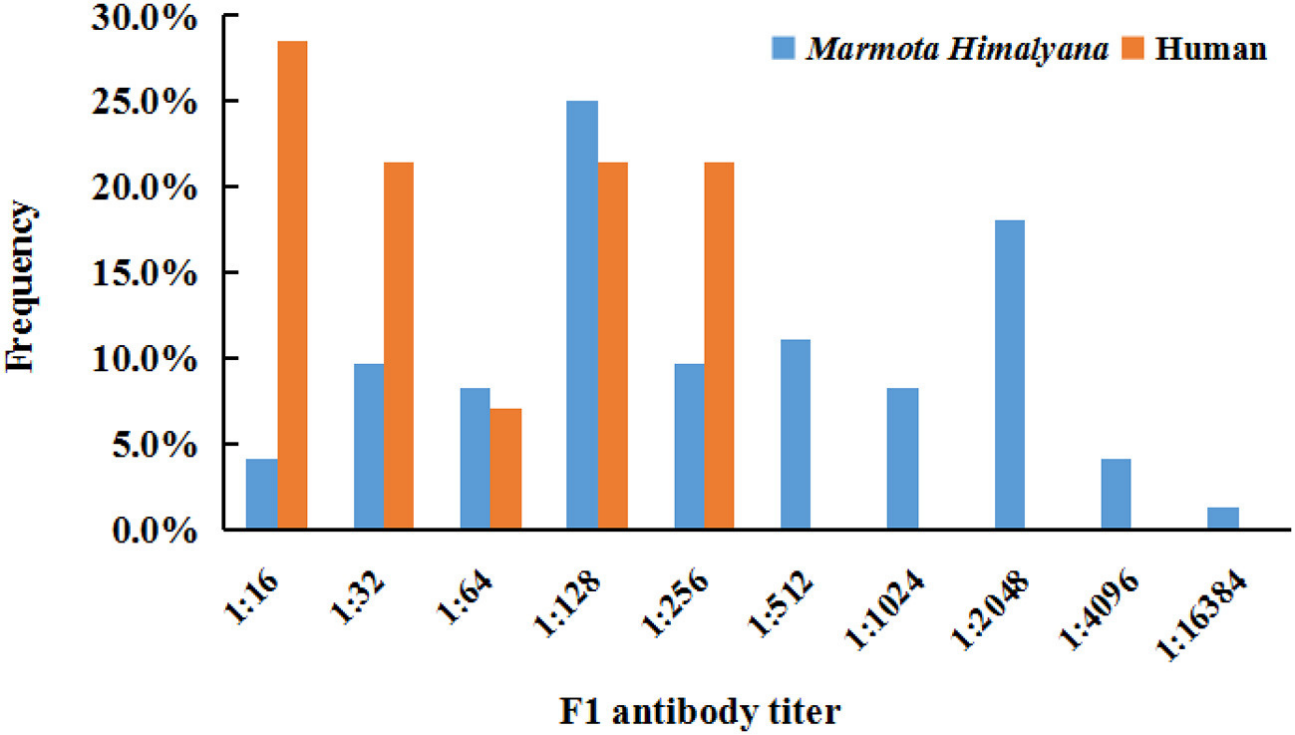
Frequency of *Yersinia pestis* F1 antibody titer in seropositive humans and *Marmota himalayana*.

The seroprevalence for plague in marmots was 25.1% (72/287). Among 72 positive samples, the titers accounting for the top two largest proportions were 1:128 (25.0%, 18/72) and 1:2,048 (18.1%, 13/72), the highest titer was 1:16,384 (1.4%, 1/72), and the lowest titer was 1:16 (4.2%, 3/72) (Figure 2). The seroprevalence for human plague infection was 3.2% (14/432) (Table 1). All of the 14 seropositive individuals were identified as previous plague infection cases. The highest titer was 1:256, accounting for 21.4% (3/14), and the lowest titer was 1:16, accounting for 28.6% (4/14) (Figure 2). Occupations with the highest seroprevalence were veterinarians (6.8%, 4/59) and herders (3.6%, 6/167).

**Table 1:** Seroprevalence of brucellosis and plague in studied human populations.

	**Brucellosis, Seroprevalence, 95% CI (Positive /Total)**	***P-*value**	**Plague, Seroprevalence, 95% CI (Positive /Total)**	***P-*value**
**Gender**		0.999		0.397
Male	1.1%, 0.2–3.1% (3/275)		2.6%, 0.7–4.3% (7/275)	
Female	1.3%, 0.1–4.5% (2/157)		4.5%, 1.2–7.4% (7/157)	
**Age group**		0.820		0.525
7~19	0.0%, 0.0–33.6% (0/9)		0.0%, 0.0–3.6% (0/9)	
20~44	1.7%, 0.3–4.7% (3/181)		2.8%, 0.4–5.0% (5/181)	
45~59	1.0%, 0.1–3.5% (2/202)		4.5%, 1.5–7.0% (9/202)	
60~70	0.0%, 0.0–8.8% (0/40)		0.0%,0.0–8.8% (0/40)	
**Occupation**		0.906		0.259
Breeder	1.8%, 0.4–5.0% (3/168)		1.8%,0.4–5.0% (3/168)	
Herder	1.2%, 0.1–4.2% (2/167)		3.6%,0.7–6.2% (6/167)	
Veterinarian	0.0%, 0.0–6.1% (0/59)		6.8%,1.8–15.5% (4/59)	
Other occupations	0.0%, 0.0–9.3% (0/38)		2.6%,0.1–13.5% (1/38)	
**Total**	1.2%, 0.2–2.1% (5/432)		3.2%,1.5–4.8% (14/432)	

The seroprevalence for livestock brucellosis infection was 4.2% (243/5,799). It was higher in camels (6.2%, 236/3,820) and sheepdogs (1.8%, 2/110) than in cattle (0.4%, 4/987) and sheep (0.1%, 1/882); the seroprevalence for marmots was 0 (0%, 0/360).

In humans, the seroprevalence for brucellosis was 1.2% (5/432) (Table 2). The titers were 1:40 for two samples, and 1:20, 1:80, and 1:160 for the other three. Occupations with the highest seroprevalence were breeders (1.8%, 3/168) and herders (1.2%, 2/167). No statistically significant differences were found in seroprevalence between different groups in plague or brucellosis infection.

**Table 2:** Brucellosis seroprevalence in different hosts.

**Host**	**Collection period**	**No. specimens**	**No. positive specimens**	**Seroprevalence (%)**
Cattle	2019–2020	987	4	0.4
Sheep	2019–2020	882	1	0.1
Camel	2019–2020	3,820	236	6.2
Sheepdog	2020–2021	110	2	1.8
Marmot	2018–2021	360	0	0.0
Human	2020–2021	432	5	1.2

## Discussion

The potential danger of animal plague prevalence should not be underestimated: one-fourth of the marmots were positive for F1-antibody, and seroprevalence for people having contact with livestock animals was 3.2%, which indicates evidence of exposure to the *Yersinia* antigen (dead or live pathogenic materials). On the other hand, the findings of F1 antibody-positive unreported cases suggests that these previous plague infection cases had been ignored or misdiagnosed on routine clinical examination. Hence, routine surveillance of plague should be strengthened as some plage infection cases could be missed on routine clinical examination.

Several reasons might explain why previous plague infections have been missed and why severe plague cases are rare in the most active regions of the *M. himalayana* plague foci of the Qinghai-Tibet Plateau, Altun Mountains. First, the risk of human transmission is low because humans live in vast, sparsely populated areas. Second, the local Centers for Disease Control and Prevention distributed plague first-aid kits (Figure 1C) for herdsmen and breeders containing tablets of oxytetracycline, sulfamethoxazole, sulfadiazine, and trimethoprim, with a reminder to take the medicine and seek prompt medical advice if fever and other typical plague symptoms develop after contacting rodents such as marmots and hares. The majority of studied people were breeders and herders, living on vast land away from hospitals (Table 1). It is not known how many of them took the medicine, but the F1 antibody-positive cases had high chances. They may have taken medicine from a first-aid kit and recovered from the plague. Third, because of propaganda and customs, most local people will not eat dead animals that are found, reducing the risk of contracting pneumonic plague. The bubonic plague caused by fleabites was likely the plague type in plague cases, which has a long incubation period, no human-to-human transmission, and low mortality (11). Drugs in the incubation period can control infection progression in the early stages, avoid deterioration in the condition, and greatly reduce the case fatality rate (18). This indicates the importance of early prophylactic medication.

Brucellosis outbreaks occurred among camels in the region where brucella seroprevalence in camels was 6.2% but that of the studied human population was only 1.2% (19, 20). The rate was lower than that in Shanxi (2.91%) and Xinjiang (1.68%), which are also areas with high brucellosis incidence (21). The relatively low rate might be due to humans having less chance of contact with livestock in pastoral areas than that in captive breeding. In addition, most of the people living in the area are Kazak ethnicity, and they reduce risk of brucella infection by practicing good hygiene, including not eating raw meat and or found dead animals, washing their hands under running water before meals, and not optional touching food when visiting as a guest.

The way camels got infected is of concern, as they are usually raised separately with other livestock in the pastoral areas. Wild marmots have chances of contact with camels, but their negative results for brucellosis suggest that this is infrequent. Therefore, marmots and other livestock were unlikely the sources of infection. Sheepdogs had the second highest brucellosis seroprevalence after that of camels. Dogs are usually affected by *Brucella canis* but can also be infected with *Brucella melitensis* which camels are highly susceptible to. Camels can have close contact with sheepdogs when grazing. It is likely that diseased camels infected the dogs and the infection is circulating in the population. There is another possibility that the sheepdogs may be the source of infection in camels. Infected camels are difficult to detect because they are nearly asymptomatic (22), which increases the possibility of mutual infection among camels as the cause of outbreaks. Brucellosis in humans and animals caused by dogs has also been reported previously (23). Dog–camel transmission as a possible cause of brucellosis outbreaks indicates that host animals with low infection rates may also become secondary or transient sources of infection. Therefore, secondary hosts also need to be considered in zoonoses prevention and control, especially in natural foci associated with developed animal husbandry.

This serological investigation confirmed the existence of missed previous infection with plague in these foci and indicated the risk of the secondary host animal in the transmission of brucellosis infection. In the investigation of the transmission chain of brucellosis infection in camels, the serological investigation has shown that sheepdogs have a higher risk of transmission than other animals. However, because of the lack of a questionnaire survey on possible risk factors, the source of camel infection can only be speculated. More detailed epidemiological exposure history and etiological analysis will be helpful to determine the risk factors and infectious chains of these two zoonotic diseases and should be confirmed in further studies.

## Data availability statement

The original contributions presented in the study are included in the article/supplementary material, further inquiries can be directed to the corresponding author.

## Ethics statement

The studies involving human participants were reviewed and approved by the Laboratory Animal Welfare & Ethics Committee of the National Institute for Communicable Disease Control and Prevention of the Chinese Center for Disease Control and Prevention. Written informed consent to participate in this study was provided by the participants' legal guardian/next of kin. The animal study was reviewed and approved by the Laboratory Animal Welfare & Ethics Committee of the National Institute for Communicable Disease Control and Prevention of the Chinese Center for Disease Control and Prevention. Written informed consent was obtained from the owners for the participation of their animals in this study.

## Author contributions

XW contributed to the conception, design of the work, and supervised the work. SQ, JL, DT, YC, DL, ZH, WW, and HH performed the experiments. SQ, DT, and RD performed the analysis and interpretation of the data. SQ, JL, YC, and HJ drafted the manuscript. RD, XL, AB, and XZ conceived the work and critically review the manuscript. All authors contributed to the article and approved the submitted version.

## Funding

This work was supported by the National Science and Technology Major Project (2018ZX10713-003-002 and 2018ZX10713-001-002).

## Conflict of interest

The authors declare that the research was conducted in the absence of any commercial or financial relationships that could be construed as a potential conflict of interest.

## Publisher's note

All claims expressed in this article are solely those of the authors and do not necessarily represent those of their affiliated organizations, or those of the publisher, the editors and the reviewers. Any product that may be evaluated in this article, or claim that may be made by its manufacturer, is not guaranteed or endorsed by the publisher.
